# A novel capitate bone Ilizarov external fixator for treating Kienböck’s disease: an anatomical and biomechanical study

**DOI:** 10.1038/s41598-024-55445-3

**Published:** 2024-03-26

**Authors:** Feifan Xiang, Wei Fan, Xiaoqi Tan, Jinhui Liu, Hao Gu, Yunkang Yang

**Affiliations:** 1 Department of Orthopedics, The Affiliated Hospital of Southwest Medical University, No 25 Tai Ping Street, Jiang Yang District, Luzhou, 646000 Sichuan China; 2 Sichuan Provincial Laboratory of Orthopaedic Engineering, Luzhou, 646000 China; 3 Department of Dermatology, The Affiliated Hospital of Southwest Medical University, Luzhou, 646000 China; 4 Department of Orthopedics, Baoji Central Hospital, 8 Jiangtan Road, Baoji, 721008 Shanxi China

**Keywords:** Osteonecrosis of lunate, Capitate bone, Capitate Ilizarov external fixator, Orthofix external fixator, Biomechanical experiments, Trauma, Medical research

## Abstract

This study aims to measure anatomical data of the capitate bone, develop an external fixator for treating late-stage osteonecrosis of lunate through Ilizarov technique, and evaluate its biomechanical performance. We selected eight wrist joint specimens to measure various parameters of the capitate bone, including its length, the distance from the junction of capitate head and body to the proximal end, as well as the width of its proximal head and distal body. Additionally, we measured these same indicators in 107 patients who had undergone wrist X-ray examination. Based on our measurements, we categorized the capitate bone into two groups and designed two types of capitate bone Ilizarov external fixator (CIEF) for it. Then, we compared it with the orthofix external fixator (OEF) through dynamic fatigue biomechanical experiments and pull-out resistance experiments. The results of the measurement revealed two categories of general patterns in the capitate bone. The first type maintains a consistent longitudinal axis between the proximal and distal ends. The second type is characterized by its proximal end being close to the radial side and its distal end being close to the ulnar side. In the dynamic tensile fatigue test, CIEF-A and CIEF-B had smaller maximum displacement values compared to the OEF (P < 0.05). In the anti-pull-out experiment, both CIEF-A and CIEF-B exhibited higher maximum pull-out force than the OEF (P < 0.05). CIFE is a treatment for advanced osteonecrosis of the lunate bone. It is specifically designed to align with the anatomical characteristics of the capitate bone, providing excellent biomechanical properties and a simple clinical procedure. However, additional clinical experiments are needed to confirm its effectiveness in the future.

## Introduction

Osteonecrosis of the lunate, also known as Kienböck’s disease, primarily affects physically active men aged 20 to 40. It has a slow onset and long duration, with initial symptoms often going unnoticed. However, as the disease advances, symptoms gradually worsen, eventually resulting in loss of wrist joint function^1^.At present, based on the different manifestations of avascular necrosis in X-rays, Lichtman staging is commonly used clinically to determine the severity of avascular necrosis^2^.

Conservative management is typically used for lunate bone necrosis before reaching Lichtman III A. However, once the disease progresses to Lichtman III B and beyond, conservative management becomes ineffective in preventing wrist bone collapse. Consequently, surgery becomes necessary^3^. In such cases, surgical options primarily involve resecting or replacing the necrotic lunate bone. Treatment for this advanced stage of the disease may include carpal osteotomy, carpal excision, wrist fusion, vascular bone grafting, lunate extraction, and artificial lunate replacement^4–9^. The impact of various treatment methods on wrist joint function varies, as do the complications they may cause. Additionally, there is currently no consensus on the best treatment approach for lunate bone necrosis. Therefore, continuous exploration and innovation in treatment methods can further enhance the effectiveness of treating lunate bone necrosis^3^.

Over the years, Ilizarov technology has been successfully used in clinical practice for various purposes. It has proven effective in treating long bone defects and osteomyelitis resulting from trauma^10,11^. Additionally, it has been utilized to restore limb length and function by lengthening phalanges or metacarpals. For instance, scholars have achieved positive outcomes by using the Ilizarov microfixator to extend amputation stumps of distal phalanges measuring less than 10 mm^12^. Similarly, the Mini–Ring Ilizarov Device has been employed to extend the thumb and metacarpal bone, with all patients experiencing satisfactory results during follow-up^13^. Currently, among all wrist bones, there are only reports on the application of this technique in treating scaphoid fractures^14,15^. However, there is no research available regarding the use of this technique for osteonecrosis of lunate.

**Figure 1:**
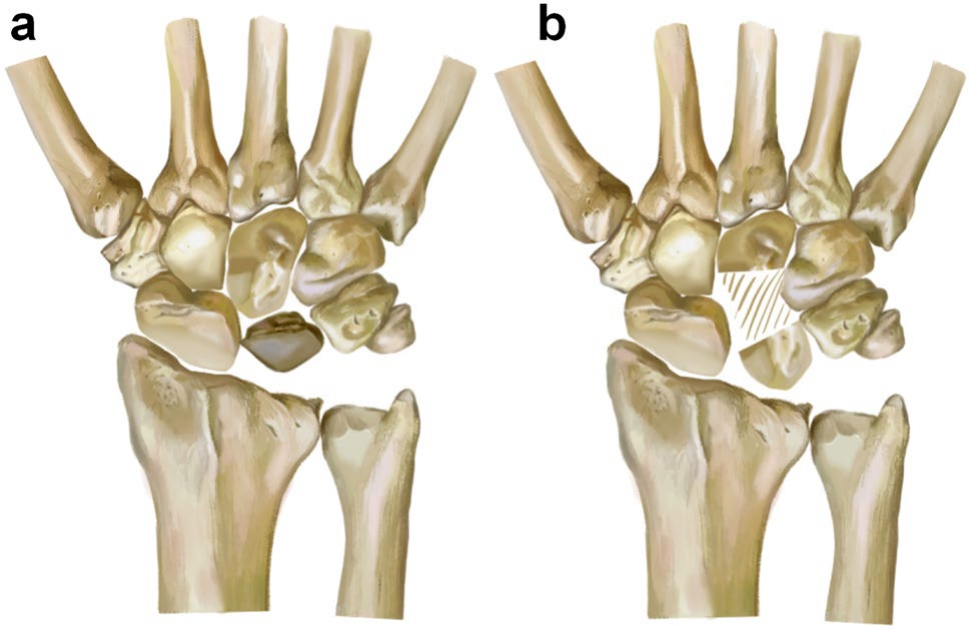
(**a**,**b**) An osteotomy is performed to lengthen the capitate bone as a replacement for the necrotic lunate bone.

The lunate bone forms a joint with the radius at its proximal end and with the capitate bone at its distal end. The palmar surface of the lunate bone is quadrilateral, while the lateral surface is crescent-shaped. Scholars utilized computer software to reconstruct and compare the anatomical morphology of the capitate bone and lunate bone. They measured various dimensions, including length, width, height, arc length, arc height, and diameter of the joint surface. The findings revealed a striking similarity between the lunate bone and the proximal end of the capitate bone^16,17^. Therefore, we hypothesize that when avascular necrosis of the lunate bone reaches stage Lichtman III B or later, after removing the necrotic lunate bone, Ilizarov technique could be applied to the capitate bone. By gradually lengthening and replacing it as a substitute for the lunate bone, we can achieve the goal of treating lunate avascular necrosis and successfully avoid complications such as wrist collapse, prosthesis loosening, and non-union after grafting caused by previous treatment methods (Fig. 1a,b) However, we have observed that the commonly used OEF in clinical practice often fails to properly align the proximal and distal ends of the capitate bone after osteotomy. As a result, this frequently leads to inadequate stability of the external fixator and prevents optimal clinical outcomes. Additionally, the micro external stents used clinically mainly fixate and lengthen phalanges and metacarpals. These stents do not align with the anatomical characteristics of capitate bone and fail to securely fixate its proximal and distal ends after osteotomy, often leading to unsuccessful lengthening attempts. Therefore, we have redesigned the external fixation device for capitate bone osteotomy lengthening by measuring relevant anatomical data of wrist joint specimens and X-rays. The redesign includes adjusting the distance between nail holes, the number and distribution of screws to conform to the anatomy of the wrist joint and capitate bone.

We will conduct clinical manipulation experiments on wrist joint specimens and use polyurethane material carriers for biomechanical testing. This will allow us to compare the performance of our newly developed CIEF with commonly used OEF. Ultimately, our goal is to provide a theoretical and scientific basis for using capitate bone osteotomy and lengthening as a treatment for lunate osteonecrosis in clinical settings.

## Materials and methods

### Measurement of capitate bone structure data and the development of CIEF

**Figure 2:**
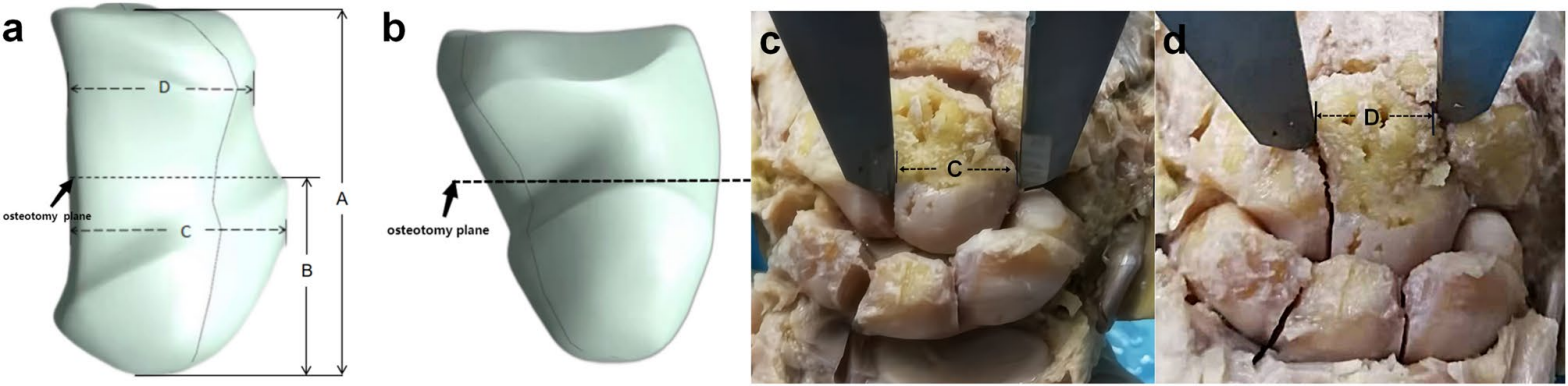
Schematic diagram of data measurement of capitate bone. (**a**,**b**) Schematic diagram of different measurement planes. (**c**,**d**) Schematic diagram of wrist joint specimen measurement.(**a**) The length of the capitate bone. (**b**) The distance from the osteotomy plane to the proximal end. (**c**) The width of proximal head of capitate bone. (**d**) The width of distal body of capitate bone.

We selected eight adult wrist joint specimens, four on the left side and four on the right side. These specimens were provided by the Department of Anatomy and Teaching Research of Southwest Medical University. All specimens showed no external injuries or abnormalities such as deformities, tumors, fractures, collapses, or dislocations in the carpal bones. After thoroughly exposing the carpal bone, we measured various distances using a vernier caliper (Fig. 2a,b):

- The length of the capitate bone (Fig. 2A).
- The distance from the junction of the head and body of the capitate bone to the proximal end of the capitate bone: the distance from the osteotomy plane to the proximal end. (Fig. 2B).
- The width of proximal head of capitate bone (Fig. 2C).
- The width of distal body of capitate bone (Fig. 2D).

107 patients who underwent wrist joint X-ray examination at the Affiliated Hospital of Southwest Medical University from July 2019 to October 2021 were selected. These patients had no abnormalities such as wrist bone fractures, tumors, collapse, dislocation, or osteoporosis. The same parameters were measured on the imaging data of these patients’ wrist joints (Fig. 2c,d). Based on the measured anatomical parameters of the capitate bone, design and create drawings for CIEF. Then, hire a manufacturer with a medical device (Sichuan Vista Medical lnstrument Co.,Ltd) production license to produce it.

### Comparative study of surgical operations using different external fixators on specimens

The CIEF experimental group and the OEF control group were established. A skilled orthopedic surgeon performed simulated surgery on the specimens using external fixation. After removing the necrotic lunate bone, cut the capitate bone at a predetermined osteotomy plane and then apply the external fixator. Then, observe and determine the precise positions of the external fixator screws, the osteotomy plane of the capitate bone, and assess the surgical procedure’s difficulty level.

### Biomechanical experiment: dynamic fatigue biomechanical experiment

Insert three screws at the proximal end of the OEF into a cylindrical polyurethane carrier, and insert two screws at the distal end into another identical polyurethane carrier, with a distance of 15 mm between the two modules. Use the same method to fix CIEF-A and CIEF-B on separate polyurethane carriers. Three types of external fixation models were fixed onto the dynamic fatigue testing machine. To simulate the traction force mechanism of the external bracket screw during capitate bone lengthening after osteotomy, we applied a load (20–60 N, sine function periodic loading) perpendicular to the osteotomy plane and longitudinally on the external bracket screw. Each model undergoes four sets of tests, with each set consisting of 500 cycles of loading. The maximum displacement of the external fixator after cyclic stretching is recorded.

### Biomechanical experiment: pull-out resistance experiments

Install the three types of external fixators (OEF, CIEF-A, CIEF-B) onto a polyurethane carrier. Then, attach them to a universal biomechanical testing machine. Set the loading speed to 5 mm/min and perform four pull-out resistance tests for each group of external fixation screws. Connect the terminal to a computer for data recording and plotting displacement-force curves. The maximum pulling-out resistance force is defined as the force corresponding to the highest point of the curve.

### Statistic analysis

The data of this experiment was analyzed using statistical software SPSS25.0. The measurement data was presented as mean ± standard deviation ($\overline{x }\pm s$). Multiple group comparisons were performed using one-way analysis of variance (ANOVA), and individual group comparisons were conducted using the LSD-t-test. A statistically significant difference is indicated by P < 0.05.

### Ethics approval and consent to participate

This study conforms to the provisions of the Declaration of Helsinki and has been reviewed and approved by the Ethics Committee of Affiliated Hospital of Southwest Medical University (KY2023321). Informed consent was obtained from the volunteer.

## Results

### Observation of the gross morphology and measurement of structure in the capitate bone

**Figure 3:**
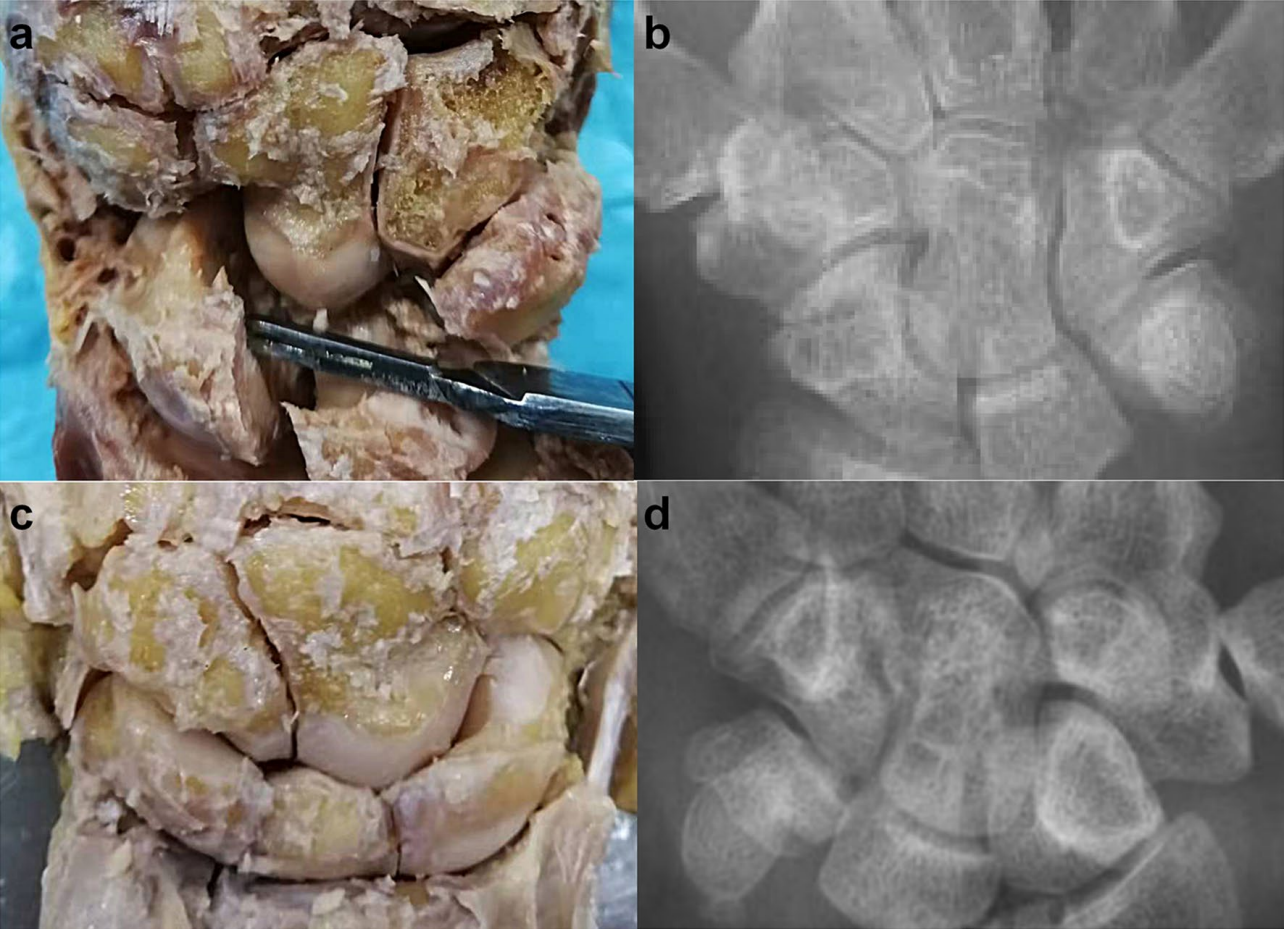
Schematic diagram of two types of capitate bone. (**a**,**b**) The first type has proximal and distal parts that generally align along the same longitudinal axis. (**c**,**d**) The second type has a proximal end inclined towards the radial side, and a distal end inclined towards the ulnar side.

We observed the gross morphology of 8 cadaveric wrist joint specimens and 107 patient wrist joint X-ray images. We identified two types of capitate bones: one type had a consistent alignment along a single longitudinal axis (Fig. 3a,b), while the other type had a proximal end deviating towards the radial side and a distal end deviating towards the ulnar side(Fig. 3c,d). Out of the 8 cadaveric specimens, one case (14%) belonged to the first type and seven cases (86%) belonged to the second type (Table 1). Amongst the 107 patient X-ray images, seventeen cases (16%) belong to the first type and ninety cases (84%) belong to second type (Table 2).The analysis of capitate bone measurements, including length, distance from the cutting plane to the proximal end, proximal width, and distal width in general specimens and radiographic images, showed no anatomical differences between the left and right wrists.

**Table 1:** Analysis and comparison of measurement data of gross specimen of capitate bone(n = 8, $\overline{x }\pm s$, mm).

Parameters	Left side	Right side	t value	P value
A	24.92 ± 0.89	23.83 ± 1.58	1.21	0.29
B	13.15 ± 0.54	12.43 ± 0.46	2.04	0.09
C	13.09 ± 1.77	13.19 ± 1.22	0.10	0.93
D	13.67 ± 1.80	13.33 ± 1.23	0.31	0.77

(A) The length of the capitate bone. (B)The distance from the osteotomy plane to the proximal end. (C) The width of proximal head of capitate bone. (D) The width of distal body of capitate bone. A statistically significant difference is indicated by P < 0.05.

**Table 2:** Analysis and comparison of measurement data of wrist X-ray imaging of capitate bone(n = 107, $\overline{x }\pm s$, mm).

Parameters	Left side	Right side	t value	P value
A	23.38 ± 2.13	23.08 ± 2.21	0.71	0.48
B	12.80 ± 1.13	12.79 ± 1.34	0.03	0.97
C	13.76 ± 1.55	13.67 ± 1.36	0.31	0.75
D	14.62 ± 1.65	14.68 ± 1.81	0.20	0.84

(A) The length of the capitate bone. (B) The distance from the osteotomy plane to the proximal end. (C) The width of proximal head of capitate bone. (D) The width of distal body of capitate bone. A statistically significant difference is indicated by P < 0.05.

### The development of CIEF: the structure and relevant parameters of CIEF

**Figure 4:**
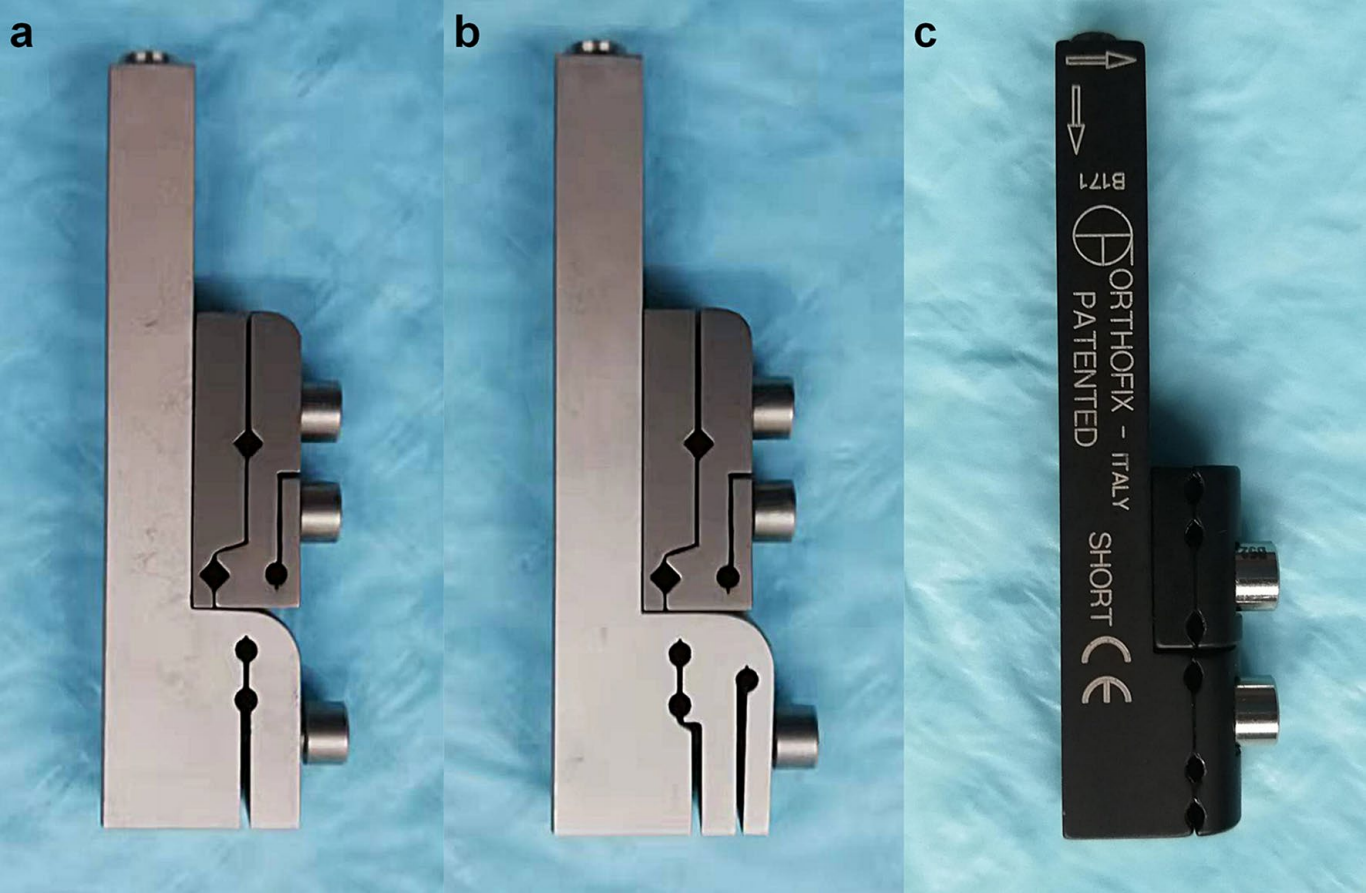
Physical diagrams of the newly developed CIEF and the original OEF. (**a**) CIEF-A. (**b**) CIEF-B. (**c**) OEF. *CIEF* capitate bone Ilizarov external fixator, *OEF* orthofix external fixator.

The structure of CIEF is designed based on anatomical measurement data of the capitate bone. Two types of external supports, CIEF-A and CIEF-B (Fig. 4a,b), are designed according to the general morphology of the capitate bone.The main shaft of CIEF is a rectangular cuboid measuring 70 mm in length, 10 mm in thickness, and with a maximum extension of 16 mm. The nail holes are designed to have a diameter of 2 mm.

The head of the CIEF serves as a fixation module, which is responsible for fixing the proximal end of the capitate bone. For the CIEF-A, there are two longitudinal screws on the same axis as the fixed module. On the other hand, for the second type called CIEF-B, there is an eccentric triangle bracket screw hole in its fixed module.The CIEF fixation module measures 20 mm in length and 10 mm in width. The distance between the two nail holes of CIEF-A is 5 mm, and the distance from the nail hole to the edge of the module is 3 mm. For the CIEF-B fixation module, there are three nail holes. The two longitudinal nail holes on the ulnar side are located 1.5 mm away from the central axis. Additionally, there is a radial nail hole that is situated 4.5 mm away from the central axis.

The sliding modules of CIEF-A and CIEF-B are identical, measuring 27 mm in length and 10 mm in width. Additionally, there are two near-end holes symmetrically distributed along the central axis, with a distance of 6 mm between them. This particular module fixes both the distal end of the capitate bone and the third metacarpal bone simultaneously while sliding along with bolt rod extension.

### Comparison of surgical procedures on gross specimens: CIEF VS OEF

The OEF consists of two modules, each with three nail holes symmetrically distributed. The adjacent nail holes are spaced 4 mm and 8 mm apart (Fig. 4c). Initially, we selected the two screw holes closest to each other to secure the proximal end of the capitate bone. However, we discovered that only one screw could be used in the other module to fix the distal end of the capitate bone. Regardless of whether we choose an osteotomy plane at the proximal and distal junction or near the base of the capitate bone, it does not provide sufficient stability.

**Figure 5:**
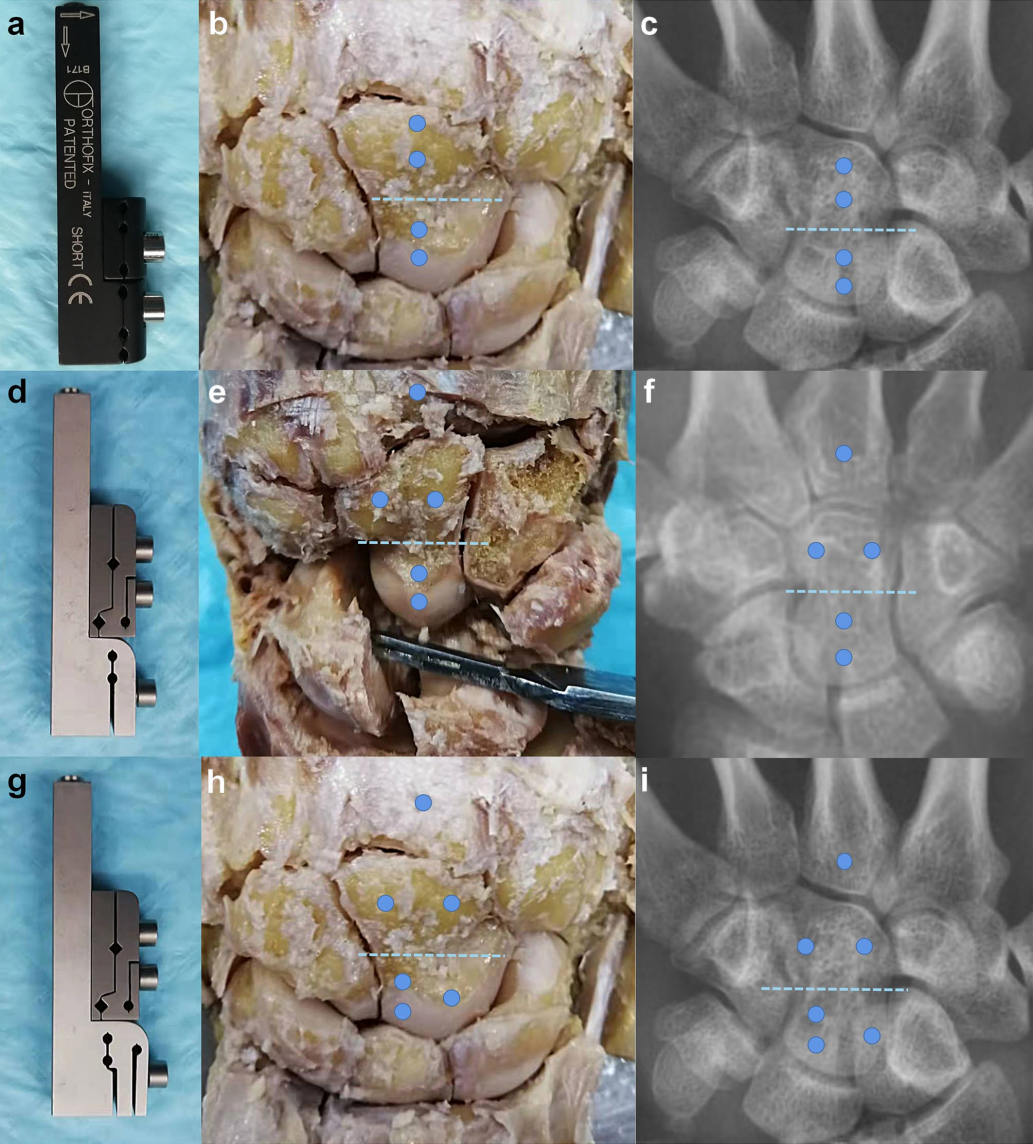
Schematic diagram of needle insertion position and osteotomy plane of different external fixators. Dark blue dot represents needle insertion position, and Baby blue dotted line represents osteotomy plane. (**a**–**c**) OEF. (**d**–**f**) CIEF-A. (**g**–**i**) CIEF-B. *CIEF* capitate bone Ilizarov external fixator, *OEF* orthofix external fixator.

Next, we chose two screws with a greater distance to secure the proximal end of the capitate bone. However, we discovered that when we used the junction between the proximal and distal ends of the capitate bone as the osteotomy plane, it was not possible to effectively implant the screws into the proximal end due to their long spacing. As a result, both screws ended up being positioned very close to both the osteotomy plane and the proximal articular surface of the capitate bone. When the osteotomy plane is positioned near the distal end of the capitate bone, only one screw can be used to fixate the distal end of the capitate bone. Consequently, regardless of which osteotomy plane or screw configuration is chosen, we have observed that OEF cannot effectively and reliably stabilize the Capitate bone after osteotomy (Fig. 5a–c).

We used the same experimental method to apply CIEF to wrist joint specimens. Our findings indicate that CIEF screws are effective in fixing both the proximal and distal ends of the capitate bone. The placement of the screws aligns with the osteotomy plane and the proximal and distal joint edges of the capitate bone, ensuring secure fixation. (Fig. 5d–i).

### Biomechanical experiments

**Figure 6:**
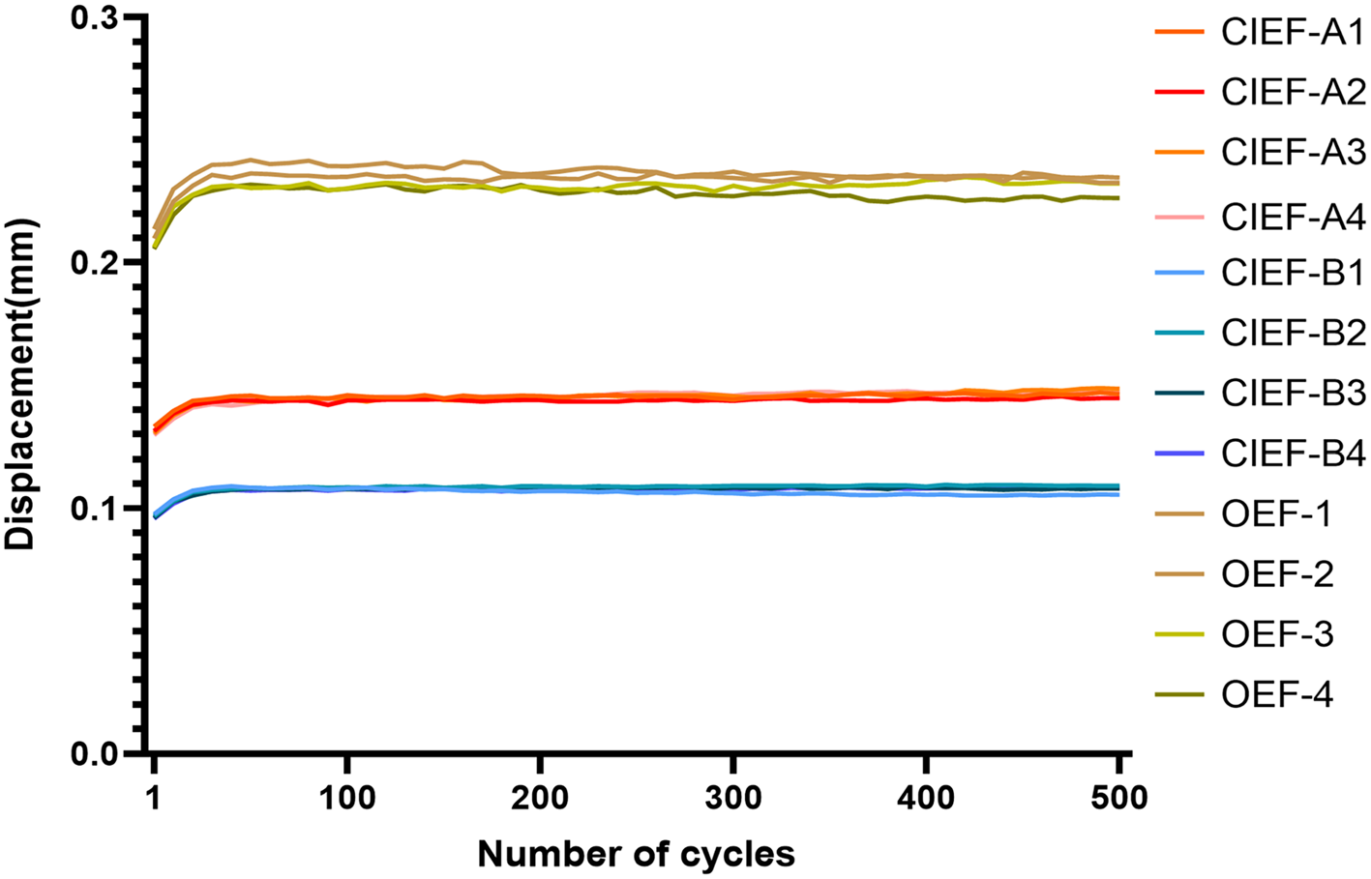
Schematic diagram of displacement for different groups after 500 cycles. *CIEF* capitate bone Ilizarov external fixator. *OEF* orthofix external fixator.

The dynamic fatigue test results indicate that both CIEF-A and CIEF-B had smaller displacements than OEF after cyclic loading (P < 0.01). Specifically, the average displacement of the CIEF-A group decreased by 37.3% (0.252 ± 0.32 mm vs 0.158 ± 0.24 mm), while the average displacement of the CIEF-B group decreased by 53.5% (0.252 ± 0.32 mm vs 0.117 ± 0.16 mm). This suggests that CIEF has better tensile performance compared to OEF (Fig. 6).

In pull-out resistance experiments, both CIEF-A and CIEF-B exhibited higher maximum pull-out forces compared to the OEF group. Specifically, the maximum pull-out force of CIEF-A increased by 30.3% (0.622 ± 0.09 KN vs 0.811 ± 0.53 KN), while that of CIEF-B increased by 63.1% (0.622 ± 0.09 KN vs 1.015 ± 0.81 KN). These results clearly demonstrate the superior stability of CIEF over OEF (Table 3).

**Table 3:** Single factor analysis of variance on the experimental data of pullout test of three groups of external bracket screws(n = 5, $\overline{x }$±s, KN).

Group	Pull-out force
OEF	0.622 ± 0.090
CIEF-A	0.811 ± 0.533
CIEF-B	1.015 ± 0.813
F value	32.957
P value	< 0.01

*OEF* orthofix external fixator, *CIEF* capitate bone Ilizarov external fixator. A statistically significant difference is indicated by P < 0.05.

## Discussion

To accurately develop the CIEF, we re-measured the relevant anatomical data of the capitate bone. However, we found slight discrepancies compared to previous research results. For instance, scholars like Wu Huan have used micro CT and computer software to reconstruct the blood supply and morphology of the capitate bone. According to their findings, the length and width of the capitete bone measured by computer software are 16.30 ± 0.43 mm and 9.75 ± 0.76 mm respectively^17^. This differs significantly from the measurements obtained in our experiment, and we believe this may be because computer reconstruction cannot effectively rebuild the edge bone quality of the capitate bone, while measurements taken from gross specimens include cartilage thickness at both proximal and distal joint surfaces of the capitate bone. Therefore, anatomical values measured on physical specimens are larger. In clinical surgical operations, it is necessary to consider the osteotomy plane of the capitate bone as well as specific screw placement for external fixation, while also trying to avoid damaging cartilage at its proximal end. Therefore, we believe that an external fixation design based on gross specimen data combined with wrist X-ray imaging measurement of the capitate bone is more reasonable and suitable for clinical application.

Regarding the blood supply situation of the capitate bone and the selection of osteotomy planes, Kadar et al.^18^ scholars observed the intraosseous blood supply of the capitate bone through high-resolution microcomputed tomography imaging technology. When analyzing the relationship between fracture lines of the capitate bone and its nutrient vessel entry points, they found that most vessels enter the distal end of the capitate bone and supply in a retrograde manner towards the proximal end. Additionally, in most specimens, at least one vessel enters directly into the proximal end through palmar side ligaments to provide direct supply to it. Therefore, we believe that besides retrograde supply from the distal end, the blood supply source of the proximal end of the capitate bone may also include direct supply to the proximal end. Research has shown that most of the nourishing blood vessels of the capitate bone are distributed approximately 2 mm away from its base^19^. Scholars have also studied the distribution of nourishing foramina in the capitate bone and found that they are mainly concentrated in the middle and distal one-third^20,21^. Therefore, based on anatomical and blood supply considerations, we position our osteotomy plane at a narrow section where it intersects between near and far ends of the capitate bone, slightly closer to its far-end. This plane is chosen primarily because it represents a distinct anatomical landmark at a narrow junction between head and body parts, making it easier to identify during clinical procedures. The osteotomy plane is positioned slightly closer to the distal end of the capitate bone for two reasons. Firstly, while there is direct blood supply entering the proximal end of the capitate bone, a significant concentration of rich blood supply remains in the distal end. Therefore, being slightly closer to the distal end helps preserve more blood supply in the proximal end of the capitate bone. Secondly, by performing an osteotomy slightly closer the distal end, we can ensure that intramembrane osteosynthesis size aligns with the wrist anatomy. This promotes better recovery of wrist joint function and maintains consistency with the structure at its distal end.

In orthopaedics, screws are commonly used as implants. However, before these screws can be clinically applied, their performance on human bones needs to be tested. Obtaining human bones for testing purposes is difficult due to scarcity. To overcome this challenge, foreign scholars have developed polyurethane foam materials that closely resemble human bones and possess consistent mechanical properties. These foam plastics simulate the structure of bone trabeculae, cancellous bone, and cortical bone, allowing them to replicate the mechanical properties of human bones. The stress–strain curve of these materials closely resembles that obtained from studying the inherent connection of human bones, and their elastic modulus falls within the range of human bones^22,23^. In biomechanical experiments involving new screws, some scholars often use polyurethane materials as test carriers^24^. In the preliminary biomechanical tests, we discovered that the density of the capitate bone decreased when using preserved cadaver specimens as carriers. This led to difficulties in securely fixing external support screws to the capitate bone, which had a significant impact on our experimental results. As a result, for this experiment, we will be using polyurethane foam instead of wrist joint specimens as test carriers to investigate the biomechanical properties associated with CIEF and OEF.

To meet fixation requirements during the bone lengthening process after osteotomy, two fixed screws are designed along the central axis in the fixation module of CIEF-A for type I capitate bones. This facilitates both fixation and subsequent lengthening. For type II capitate bones, the nail holes in the fixation module of CIEF-B are distributed in an eccentric triangular pattern to achieve greater stability. Our biomechanical experimental results confirm that CIEF-B exhibits superior overall performance due to its triangular distribution of screws in both the fixation and sliding modules, providing stronger stability. In contrast, only the nail tracks in the fixation module of CIEF-A show a triangular distribution, which may explain its slightly inferior biomechanical characteristics compared to CIEF-B. Additionally, the screws of OEF are aligned in a straight line along the external fixator’s longitudinal axis, rather than achieving a stable triangular distribution. This lack of triangular stability may be the reason for OEF’s inferior biomechanical characteristics.

This study has several limitations. Firstly, the sample size for each group in this experiment is relatively small. To obtain more authentic and complete data, it may be necessary to increase the sample size in future stages. Secondly, we used polyurethane material with a density close to cancellous bone as the test carrier instead of capitate bone specimens. While polyurethane foam closely resembles cancellous bone density, it does not fully simulate all characteristics of natural cancellous bone. Therefore, these findings should be considered as reference only, and further testing and research on fresh wrist joint cadaver specimens are needed in later stages. Lastly, although CIEF was designed and produced for the first time, there have been no clinical operation experiments conducted yet. Further multicenter prospective randomized clinical trials are still required to confirm its clinical efficacy.

## Conclusion

This study designed a special external stent for capitate bone osteotomy and lengthening. The design of CIEF was based on anatomical data of the capitate bone, filling the gap in external fixation for capitate bones. This external fixator offers excellent biomechanical performance and easy clinical operation, making it a potential new treatment for advanced lunate bone necrosis. However, further clinical studies are required to confirm its therapeutic effects.

## Data Availability

The data used and/or analyzed during the current study are available from the corresponding author upon reasonable request.
